# The Clinical Impact of Antibiotic Allergy Labels on One‐Year Outcomes of Solid Organ Transplant Recipients

**DOI:** 10.1111/ctr.70498

**Published:** 2026-03-02

**Authors:** Sashi Niranjan Nair, Paul Bigliardi, Lauren Fontana

**Affiliations:** ^1^ Division of Infectious Diseases Department of Medicine University of Minnesota Medical School Minneapolis Minnesota USA; ^2^ Division of Dermato‐Allergy Department of Dermatology University of Minnesota Medical School Minneapolis Minnesota USA

**Keywords:** anti‐bacterial agents, Clostridium, hypersensitivity, organ transplantation

## Abstract

Antibiotic allergy labels (AALs) are common and often incorrect. They have many potential impacts, including the use of broader‐spectrum antibiotics and suboptimal treatment of infections. The impact of inaccurate allergy labels on post‐transplant outcomes in the solid organ transplant population is not well described. We performed a retrospective review of 2,373 consecutive solid organ transplants occurring between 2011 and 2021, to analyze the impact of AALs, specifically penicillin, on outcomes in the first year after transplantation. Three hundred and twenty‐two patients (13.6%) had a penicillin allergy label, while 572 patients (24%) had at least one antibiotic allergy label. Patients with a penicillin allergy label were more likely to have a positive *Clostridioides difficile* test (*p* = 0.021). Patients with allergy labels also had significantly more utilization of alternative antimicrobial agents (*p* < 0.001) and longer inpatient hospital durations (*p* = 0.013). This study suggests that AALs may be a risk factor for inferior outcomes after solid organ transplantation and could represent a modifiable target for pre‐transplant optimization.

**Abbreviations**
AAL: antibiotic allergy label
CDI: *Clostridioides difficile* infection
DOT: days of therapy
IV: intravenous
PAL: penicillin allergy label
SOT: solid organ transplantation

## 1 Introduction

Antibiotic allergy labels (AALs), specifically penicillin allergy labels (PALs), are prevalent yet frequently inaccurate. The prevalence of PALs ranges from 6%–25%, with the highest rates recorded in patients admitted to the hospital [1, 2]. However, greater than 90% of these patients are found to be non‐allergic when verified through skin testing or drug challenge [3]. This is due to a variety of factors, including historical manufacturing practices, decreasing IV penicillin utilization, waning allergenicity over time, and side effect or allergy misattribution [4, 5, 6, 7].

Although PALs naturally lead to penicillin avoidance, they often trigger broader, unnecessary avoidance of all β‐lactams by prescribers, despite low rates of cross reactivity [8]. Compared to β‐lactam antibiotics such as penicillins or cephalosporins which are often first‐line agents, alternative antibiotics such as fluoroquinolones, carbapenems and vancomycin are associated with significant complications. These include the emergence of drug‐resistant organisms, a higher risk of *Clostridioides difficile* infection (CDI), higher costs and duration of inpatient hospitalizations, and increased failure of antibiotic therapy [8, 9, 10, 11, 12]. These complications can be mitigated by the removal of inaccurate AALs, or ‘de‐labeling’. While the safety and benefits of de‐labeling programs (i.e., cost savings and reduced length of stay) are well documented in the general population, data in specialized populations remain sparse [13].

Solid‐organ transplant (SOT) recipients represent a uniquely high‐risk population due to their immunocompromised state. Although SOT outcomes have improved over time infections, specifically those caused by antibiotic‐resistant organisms and CDI, remain a major cause of morbidity and mortality [14, 15, 16, 17, 18]. SOT recipients utilize antibiotics more frequently and possess higher rates of AALs than the general population [19, 20, 21, 22]. Despite this, the impact of AALs on clinical outcomes in the SOT population remains poorly defined. Existing studies, which have focused on the very early post‐transplant period, suggest that β‐lactam allergy labels increased alternative antibiotic use but were unable to define an impact on clinical outcomes including mortality, CDI, or the duration of hospitalization [21, 22].

CDI is a particular concern in SOT with a 5‐10 percent prevalence and a hospital onset incidence 5 times higher than the general population [23, 24]. AALs exacerbate this risk by necessitating alternative agents which are strongly associated with CDI risk. Given CDI is associated with up to a threefold increase in mortality, prolonged hospital stays, and higher healthcare costs, understanding the role of AALs in this context is critical [25, 26]. In this context we hypothesized that AALs, specifically PAL may represent an independent risk factor for adverse outcomes, particularly CDI. Here, we describe the prevalence of AALs in SOT recipients at our institution and evaluate their impact on one‐year clinical outcomes.

## 2 Methods

### 2.1 Study Setting, Design and Population

We performed a retrospective cohort study, including patients undergoing their first single organ transplant at the University of Minnesota over a nine‐year period between 1/1/2012 and 1/1/2021. The University of Minnesota is a multiorgan transplant center that performs and provides pre‐ and post‐ operative care for heart, lung, liver, kidney, pancreas, small bowel and multiorgan transplant candidates and recipients. Data from the first year following transplantation was reviewed. Patients aged less than 18, undergoing a second or combined transplant (other than pancreas along with kidney) or those who opted out of research were excluded. All adult single organ recipients without exclusion criteria were included. This study was approved by the University of Minnesota IRB and adhered to ethical principles of research as outlined in the declaration of Helsinki.

### 2.2 Data Sources, Study Definitions and Outcomes

Our institutional database consists of major demographic, clinical, and outcomes data and is prospectively maintained by analysts to obtain longitudinal data on patients. Key clinical and quality metrics such as duration and number of hospitalizations, death and graft loss in the first year after transplant is specifically captured. Graft loss was defined as per UNOS criteria [27]. Data on key baseline (pretransplant) co‐morbidity rates of diabetes, ascites, chronic obstructive pulmonary disease, end stage renal disease, obesity and cystic fibrosis were additionally obtained from the database. Allergy labels present on the date of transplantation were obtained from the medical record and categorized into antibiotic classes by an infectious disease trained physician. Data regarding CDI testing was obtained from the medical record and dually sourced from an infection prevention database, and any positive *C. difficle* polymerase chain reaction (PCR) test occurring in the first year after the date of transplantation was included. *C. difficle* PCR testing during the entire study period was performed on the Cepheid Xpert platform (Cepheid, Sunnyvale, California). *C. difficile* testing at our institution has been regulated by institutional practice guidelines and mandatory electronic medical record checks, reserving it for only symptomatic patients who do not have an alternative explanation for diarrhea, such as recent stool softener use (Supplementary Data Appendix A). Inpatient antibiotic usage data for the first year after transplant was also obtained from the medical record, including drug, dose, and duration.

Our primary clinical outcome of interest was CDI in the first year after transplant. We chose *C. Difficle* PCR test positivity as a surrogate marker for CDI given our institutional protocols limiting testing only to patients with a high pre‐test probability for true CDI. Secondary clinical outcomes included mortality and graft loss in the first year after transplant, the duration of the index hospitalization in which the transplant occurred, the total number of inpatient days in the first year after transplantation, and the total inpatient days of antibiotic therapy for fluoroquinolones, carbapenems, vancomycin and aztreonam.

### 2.3 Statistical Analysis

Data was analyzed using SPSS version 26.0 (IBM, Armonk, New York). Variables with any missing data were not included in our analysis. The baseline characteristics of the cohort were compared using Pearson's χ^2^ test for categorical variables and Mann‐Whitney‐U test for continuous variables given the non‐normal distribution. To determine associations, bivariate (χ^2^and *t*‐test) and multivariate (logistic and linear) regression analysis were performed on prespecified categorical and continuous variables respectively. Co‐variates used for all multivariable analysis were transplant type, age at time of transplant, gender, pre‐transplant diabetes and pre‐transplant obesity. These covariates were selected based on prior literature, clinical justification, and/or a p value of less than 0.05 in the bivariate analysis [28, 29]. All models were checked for multicollinearity using variance inflation factors. We performed survival analysis using the Kaplan–Meier method to estimate time‐to‐event outcomes. A *p*‐value of less than 0.05 was considered significant.

## 3 Results

### 3.1 Study Population and Demographics

We obtained data from 2,373 consecutive SOT recipients between 2012 and 2021. The baseline characteristics and demographics of this cohort are shown in Table 1. The most common AAL was penicillin, seen in 322 (13.6%) patients. In our cohort 572 (24.1%) patients had 1 AAL and 161 (6.8%) had >1 AAL (Table 1). Patients with a PAL were statistically more likely to be female, have received a lung transplant, and be obese pre‐transplant. There was no significant difference between rates of diabetes or age for patients with or without a PAL.

**TABLE 1:** Demographics and baseline characteristics with PAL association.

Patient characteristic	Overall (*N* = 2373)	No‐PAL (*N* = 2051 86.4% of total)	PAL (*N* = 322, 13.6% of total)	*p*‐value
Age, Mean (SD)	54.2 years (13.3)	54.2 years (13.2)	54.8 years (13.5)	0.393
Gender, No. (%)	906‐ Female 38.2% 1467‐ Male 61.8%	752 (36.6%) Female	154 (52.2%) Female	**< 0.001**
**Transplant type, No. (% of total)**				
Kidney	1080 (45.5%)	951 (46.4%)	129 (40.1%)	
Liver	599 (25.2%)	520 (25.4%)	79 (24.5%)	
Lung	408 (17.2%)	331 (16.1%)	77 (23.9%)	**0.014**
Heart	216 (9.1%)	187 (9.1%)	29 (9.0%)	
Pancreas	70 (2.9%)	62 (3.0%)	8 (2.5%)	
**Comorbidities, No. (% of total)**				
Pre‐transplant diabetes	753 (31.7%)	642 (31.3%)	111 (34.5%)	0.274
Pre‐transplant obesity	82 (3.5%)	64 (3.1%)	18 (5.6%)	**0.024**
**Allergy label, No. (% of total)**				
Penicillin	322 (13.6%)			
Quinolone	101 (4.3%)			
Cephalosporin	95 (4.0%)			
Macrolide	68 (2.9%)			
Vancomycin	59 (2.5%)			
Sulpha	45 (1.9%)			
Tetracycline	32 (1.3%)			
Antifungal or antiviral	21 (0.9%)			
Other antimicrobials^a^	98 (4.1%)			
More than one antimicrobial allergy	572 (24.1%)			

Abbreviations: No, Number of; S.D, Standard Deviation.

^a^ Details of the other antibiotic allergy labels are available in the supplemental materials—Appendix B.

### 3.2 Associations Between Penicillin Allergy Label and Clinical Outcomes

#### 3.2.1 Primary Outcome: *Clostridioides difficile* PCR Positivity as a Surrogate for CDI

The results of bivariate and multivariate analyses for the association between pre‐transplant PAL and recipient outcomes are shown in Table 2. Having a PAL was strongly associated with C. *difficile* PCR positivity in the post‐transplant period. The significance of the association between a positive *C. difficile* PCR and the presence of a PAL persisted in the multivariate model. Those with a PAL had an odds ratio of 1.54 for having a positive *C. difficile* PCR in this model (95% CI 1.07–2.22, *p*‐value = 0.021). *C. difficile* PCR positivity by itself was not associated with a statistically significant increase in death (OR 1.30, CI of 0.77–2.2, *p* = 0.30) or graft loss (OR 1.36, CI 0.83–2.21, *p* = 0.21) in the first year.

**TABLE 2:** Univariate and multivariate analysis of penicillin allergy and one‐year primary and secondary outcomes.

			Univariate analysis			Multivariate analysis		
Clinical outcomes	No‐PAL (*N* = 2051)	PAL (*N* = 322)	OR	95% CI	*p*‐value	OR	95% CI	*p*‐value
Positive *C. Difficle* PCR	170 (8.2%)	43 (13%)	1.71	1.19–2.44	**0.005**	1.54	1.07–2.22	**0.021**
Death	127 (6.2%)	25 (7.7%)	1.28	0.82–1.99	0.272	1.16	0.73–1.85	0.519
Graft failure	144 (7%)	29 (9%)	1.31	0.86–1.99	0.205	1.22	0.79–1.87	0.371

Antibiotic usage	Average days (S.D.)	Average days (S.D.)	Coefficient	95% CI	*p*‐value	Coefficient	95% CI	*p*‐value
Quinolone‐ D.O.T	2.469 (6.103)	4.134 (7.221)	1.67	0.93–2.40	**< 0.001**	1.57	0.83–2.31	**< 0.001**
Carbapenem‐ D.O.T	2.337 (8.508)	5.382 (10.899)	3.05	2.00–4.089	**< 0.001**	2.99	1.95–4.03	**< 0.001**
Vancomycin‐ D.O.T	4.192 (8.737)	6.149 (11.335)	1.96	0.88–3.03	**< 0.001**	1.93	0.86–3.00	**< 0.001**
Aztreonam‐DOT	0.18 (1.82)	0.43 (5.12)	0.256	−0.04–0.55	0.091	0.27	−0.30–5.68	0.078
**Hospitalization**								
Total days—index hospitalization	17.0 (23.69)	22.12 (34.10)	5.129	2.15–8.11	**0.001**	5.29	2.30–8.27	**0.001**
Total inpatient days in first year	24.24 (33.49)	29.89 (42.56)	5.64	1.55–9.74	**0.007**	5.11	1.07–9.16	**0.013**

Abbreviations: D.O.T, Days of Therapy; S.D, Standard Deviation.

#### 3.2.2 Secondary Outcomes

##### 3.2.2.1 Death and Graft Failure

Death and graft failure trended higher in those with a PAL compared with those without, but it was not statistically significant in either the univariate or multivariate analysis (Table 2). The presence or absence of PAL resulted in no difference in the timing of first year death or graft failure among those patients in which this outcome occurred.

##### 3.2.2.2 Antibiotic Usage

Measured alternative antibiotic use was significant in this cohort, with 1222 (51.5%) receiving vancomycin, 1040 (43.8%) receiving a fluoroquinolone, 509 (21.4%) a carbapenem and 33 (1.4%) Aztreonam. Among the 322 PAL patients there was significantly increased inpatient utilization of alternative antibiotics in the first year after transplant. In the univariate and multivariate analysis, we reviewed quinolone, carbapenem, vancomycin, and aztreonam days of therapy (DOT) (Table 2). In the univariate and multivariate analyses quinolone, carbapenem, and vancomycin had statistically significant longer DOT in those with a PAL. Among the antibiotic groups reviewed, carbapenems were associated with the most significant increase in DOT with a mean increase of 3.05 days (95% CI 2.0–4.08 days) in those with a PAL compared to those without. This result remained consistent in the multivariable analysis (OR 2.99, 95% CI 1.95–4.03, *p*‐value < 0.001).

##### 3.2.2.3 Hospitalization Within the First Year After Transplant

PAL was associated with a statistically significant longer index hospitalization and a greater amount of time spent in the hospital within the first year after transplant in the univariate and multivariate analyses (Table 2). The effect was strongest on the duration of the index hospitalization, accounting for a mean increase of 5.2 additional days in our multivariable model (OR 3.48, 95% CI 2.30–8.27, *p* = 0.001). This effect persisted for the entire first year with an additional 2.5 days spent in the hospital on average (95% CI 1.07–9.15, *p* value = 0.013).

### 3.3 Supplementary Analyses

#### 3.3.1 Analysis of Other Allergy Categories

Comparable results were seen when analyzing patients with a β‐lactam allergy (penicillin and/or cephalosporin and/or carbapenem), or any antibiotic allergy label versus no antibiotic allergy label (Supplementary Tables 1–4). In these supplementary analyses, a higher prevalence of allergies was seen in the lung transplant population, with significantly increased C. *difficile* PCR positivity, and alternative antibiotic usage rates and a numerical trend to higher mortality and graft loss in the first year using the same multivariable model. The effect on the duration of hospitalization persisted.

#### 3.3.2 Analysis of Trends in Variables Between 2012–2021

There were no significant changes to baseline cohort variables, antibiotic usage, CDI PCR positivity rates, hospital durations, or PAL rates during the study period (Supplementary Table 5)

## 4 Discussion

Our study adds to the growing body of literature by highlighting the potential negative impact of AALs on transplant outcomes. To our knowledge this is the largest study with the longest follow‐up to assess the cumulative impact of multiple antibiotic courses during the highest‐risk first year after transplant. By considering the entirety of this critical period, we were able to demonstrate several associations with negative clinical impacts on C. *difficle* PCR positivity, antibiotic utilization and the duration of hospital stays. Our longer follow‐up period may have enabled us to capture the downstream effects of penicillin avoidance that were not evident in prior studies with shorter follow up.

The majority of prior studies with SOT patients have had either small or mixed cohorts, some including non‐transplant patients. First in a retrospective cohort study of 313 liver transplant recipients, Khumra, et al. reported higher rates of CDI and resistant gram‐negative infections among those with antibiotic allergies [30]. In a large retrospective study utilizing de‐identified ICD‐9 codes from the national inpatient sample, involving 15,489 kidney transplant recipients Nelson, et al. reported a higher cost of hospitalization but did not find an impact on clinical outcomes [20]. The two largest prior studies, by Imlay, et al. and Zhang, et al. failed to show a significant impact of β‐lactam allergy labels on clinical outcomes such as CDI or mortality within the first 100‐180 days but highlighted similar significant impacts on antibiotic prescribing [21, 22]. These prior studies did not report data on or showed no effect of, β‐Lactam allergy labels on the duration of initial or early hospitalization. In contrast, our larger study with longer follow up and a more homogenous population was able to demonstrate an effect. Our findings could be due to the duration of follow‐up, as well as differing institutional practices, transplant protocols, and patient populations.

The high rates of AAL among our population align with this existing literature showing an increased prevalence of allergy labels in the SOT population. Similarly, our data on increased alternative antibiotic usage is in line with previously published data from other centers. Both Imlay, et al. and Zhang, et al. showed an increase in inpatient usage of alternative antibiotics in their patients with β‐lactam allergies. While these studies showed the strongest effect on aminoglycoside use with a modest effect on carbapenems, our study was slightly different, perhaps because of differing institutional practices. The significantly increased use of carbapenems in PAL patients was particularly interesting given that carbapenems themselves are also β‐lactams, highlighting the non‐evidence‐based avoidance and use of certain classes of antibiotics in patients with AALs. Our weak association between PAL and aztreonam use can perhaps be attributed to extremely low utilization of this antibiotic at our center. While we were unable to obtain specific costs, we infer that the cost would have been much larger for our PAL patients due to increased duration of hospitalization, utilization of alternative antibiotics and possible higher CDI rates. This highlights the powerful potential impact allergy assessment can have in antimicrobial stewardship programs.

We acknowledge that our study is not without limitations. First, AALs may serve as a surrogate marker of overall infectious and post operative risk. Conceivably, higher risk patients with complex pre‐transplant infectious histories have had a higher chance of accumulating AALs, compared with lower risk patients. Second, the retrospective nature of our database review restricted our analysis to discrete variables present in the electronic medical record, precluding the use of patient level datapoints. For example, despite our institutional diagnostic stewardship efforts not all positive C. *Difficle* PCR tests equate to a true infection, some would have reflected colonization. Additionally, while our penicillin allergic and nonallergic cohorts were well matched based on our available metrics, confounding variables may still remain. Furthermore given this was a single center study the findings may not be fully generalizable due to variations in practices and patient populations across institutions among other confounders. Finally, although we observed while no major shifts in antibiotic usage or baseline demographics over the study timeframe, the effect of evolving clinical practices and guidelines on individual cases across the various transplant teams could not be quantified.

## 5 Conclusion

Our findings suggest several negative clinical impacts associated with PALs in the SOT population including higher rates of C. difficile PCR positivity, longer hospitalizations, and increased use and duration of alternate antibiotics. While the retrospective nature of this study precludes a definitive causal link, this data highlights the clinical burden associated with AALs which are often inaccurate. In an era where there is continued focus on improving the outcomes of SOT patients our results propose that antibiotic allergy delabeling, specifically targeting penicillin, should be key component of the pretransplant evaluation and optimization process. Future multi‐center studies with prospective data collection, or investigation of the effect of allergy delabeling programs could validate our findings and guide efforts at improving outcomes in this high‐risk patient population.

## Funding

The authors have nothing to report.

## Disclosure

The Authors have no disclosures or conflicts of interest.

## Conflicts of Interest

The authors declare no conflicts of interest.

## Supporting information




**Table S1**: Demographics and baseline characteristics with any AAL association.
**Table S2**: Univariate and multivariate analysis of any antibiotic allergy and one‐year primary and secondary outcomes.
**Table S3**: Demographics and baseline characteristics with a BLAL association.
**Table S4**: Univariate and multivariate analysis of beta‐lactam allergy label and one‐year primary and secondary outcomes.
**Table S5**: Trends in cohort factors over time.
**Appendix A**: University of Minnesota Diarrhea/*C. Difficile* clinical decision support.
**Appendix B**: Details of “other antimicrobial” allergies.

## Data Availability

The data that support the findings of this study are available on request from the corresponding author. The data are not publicly available due to privacy or ethical restrictions.

## References

[1] E. Macy , “Penicillin and Beta‐Lactam Allergy: Epidemiology and Diagnosis,” Current Allergy and Asthma Reports 14, no. 11 (2014): 476, 10.1007/s11882-014-0476-y. 25216741

[2] L. Zhou , N. Dhopeshwarkar , K. G. Blumenthal , et al., “Drug Allergies Documented in Electronic Health Records of a Large Healthcare System,” Allergy 71, no. 9 (2016): 1305–1313, 10.1111/all.12881. 26970431 PMC12841114

[3] J. A. Trubiano , N. F. Adkinson , and E. J. Phillips , “Penicillin Allergy Is Not Necessarily Forever,” The Journal of the American Medical Association 318, no. 1 (2017): 82–83, 10.1001/jama.2017.6510. 28672303 PMC5935455

[4] E. Macy , M. Schatz , C. Lin , and K. Y. Poon , “The Falling Rate of Positive Penicillin Skin Tests From 1995 to 2007,” The Permanente Journal 13, no. 2 (2009): 12–18, 10.7812/TPP/08-073. 21373225 PMC3034425

[5] M. Blanca , M. J. Torres , J. J. García , et al., “Natural Evolution of Skin Test Sensitivity in Patients Allergic to Beta‐Lactam Antibiotics,” Journal of Allergy and Clinical Immunology 103, no. 5 Pt 1 (1999): 918–924, 10.1016/s0091-6749(99)70439-2. 10329829

[6] S. Silverman , R. Localio , and A. J. Apter , “Association Between Chronic Urticaria and Self‐Reported Penicillin Allergy,” Annals of Allergy, Asthma and Immunology 116, no. 4 (2016): 317–320, 10.1016/j.anai.2015.11.020. 26782670

[7] E. S. Shenoy , E. Macy , T. Rowe , and K. G. Blumenthal , “Evaluation and Management of Penicillin Allergy: A Review,” The Journal of the American Medical Association 321, no. 2 (2019): 188–199, 10.1001/jama.2018.19283. 30644987

[8] E. Macy and R. Contreras , “Health Care Use and Serious Infection Prevalence Associated With Penicillin “Allergy” in Hospitalized Patients: A Cohort Study,” Journal of Allergy and Clinical Immunology 133, no. 3 (2014): 790–796, 10.1016/j.jaci.2013.09.021. 24188976

[9] D. R. MacFadden , A. LaDelfa , J. Leen , et al., “Impact of Reported Beta‐Lactam Allergy on Inpatient Outcomes: A Multicenter Prospective Cohort Study,” Clinical Infectious Diseases 63, no. 7 (2016): 904–910, 10.1093/cid/ciw462. 27402820

[10] N. M. Krah , T. W. Jones , J. Lake , and A. L. Hersh , “The Impact of Antibiotic Allergy Labels on Antibiotic Exposure, Clinical Outcomes, and Healthcare Costs: A Systematic Review,” Infection Control and Hospital Epidemiology 42, no. 5 (2021): 530–548, 10.1017/ice.2020.1229. 33059777

[11] K. A. Brown , N. Khanafer , N. Daneman , and D. N. Fisman , “Meta‐Analysis of Antibiotics and the Risk of Community‐Associated *Clostridium difficile* Infection,” Antimicrobial Agents and Chemotherapy 57, no. 5 (2013): 2326–2332, 10.1128/AAC.02176-12. 23478961 PMC3632900

[12] S. H. Kim , K. H. Kim , H. B. Kim , et al., “Outcome of Vancomycin Treatment in Patients With Methicillin‐Susceptible *Staphylococcus aureus* Bacteremia,” Antimicrobial Agents and Chemotherapy 52, no. 1 (2008): 192–197, 10.1128/AAC.00700-07. 17984229 PMC2223910

[13] N. Powell , J. Stephens , D. Kohl , et al., “The Effectiveness of Interventions That Support Penicillin Allergy Assessment and Delabeling of Adult and Pediatric Patients by Nonallergy Specialists: A Systematic Review and Meta‐Analysis,” International Journal of Infectious Diseases 129 (2023): 152–161, 10.1016/j.ijid.2022.11.026. 36450321 PMC10017351

[14] A. Rana , A. Gruessner , V. G. Agopian , et al., “Survival Benefit of Solid‐Organ Transplant in the United States,” JAMA Surgery 150, no. 3 (2015): 252–259, 10.1001/jamasurg.2014.2038. 25629390

[15] N. Singh and A. P. Limaye , “Infections in Solid‐Organ Transplant Recipients,” Mandell, Douglas, and Bennett's Principles and Practice of Infectious Diseases, 8th Ed. (Elsevier, 2015): 3440–3452, 10.1016/B978-1-4557-4801-3.00313-1.

[16] P. Dorschner , L. M. McElroy , and M. G. Ison , “Nosocomial Infections Within the First Month of Solid Organ Transplantation,” Transplant Infectious Disease: An Official Journal of the Transplantation Society 16, no. 2 (2014): 171–187, 10.1111/tid.12203. 24661423 PMC12306200

[17] D. van Duin and C. van Delden , AST Infectious Diseases Community of Practice , “Multidrug‐Resistant Gram‐Negative Bacteria Infections in Solid Organ Transplantation,” American Journal of Transplantation 13, no.Suppl 4 (2013): 31–41, 10.1111/ajt.12096. 23464996

[18] J. Cowan , A. Bennett , N. Fergusson , et al., “Incidence Rate of Post‐Kidney Transplant Infection: A Retrospective Cohort Study Examining Infection Rates at a Large Canadian Multicenter Tertiary‐Care Facility,” Canadian Journal of Kidney Health and Disease 5 (2018): 2054358118799692, Published 2018 Sep 12, 10.1177/2054358118799692. 30224973 PMC6136109

[19] A. Agrawal , M. G. Ison , and L. Danziger‐Isakov , “Long‐Term Infectious Complications of Kidney Transplantation,” Clinical Journal of the American Society of Nephrology 17, no. 2 (2022): 286–295, 10.2215/CJN.15971020. 33879502 PMC8823942

[20] J. Nelson , I. Carrillo‐Martin , W. Bosch , et al., “Outcomes in Hospitalized Kidney Transplant Patients With a Penicillin Allergy Label in the United States, 2005–2014,” The Journal of Allergy and Clinical Immunology: In Practice 10, no. 3 (2022): 867–869.e2, 10.1016/j.jaip.2021.10.015. 34673288

[21] H. Imlay , E. M. Krantz , E. J. Stohs , et al., “Reported β‐Lactam and Other Antibiotic Allergies in Solid Organ and Hematopoietic Cell Transplant Recipients,” Clinical Infectious Diseases 71, no. 7 (2020): 1587–1594, 10.1093/cid/ciz1025. 31621829 PMC8241219

[22] H. L. Zhang , J. A. Anesi , K. W. Hamilton , L. Cressman , W. B. Bilker , and E. Lautenbach , “The Impact of Reported β‐Lactam Allergy on Clinical Outcomes and Antibiotic Use Among Solid Organ Transplant Recipients,” Open Forum Infectious Diseases 9, no. 8 (2022): ofac384, Published 2022 Jul 29, 10.1093/ofid/ofac384. 35983261 PMC9379814

[23] S. Paudel , I. M. Zacharioudakis , F. N. Zervou , P. D. Ziakas , and E. Mylonakis , “Prevalence of *Clostridium difficile* Infection Among Solid Organ Transplant Recipients: A Meta‐Analysis of Published Studies,” PLoS ONE 10, no. 4 (2015): e0124483, Published 2015 Apr 17, 10.1371/journal.pone.0124483. 25886133 PMC4401454

[24] J. P. Donnelly , H. E. Wang , J. E. Locke , R. B. Mannon , M. M. Safford , and J. W. Baddley , “Hospital‐Onset *Clostridium difficile* Infection Among Solid Organ Transplant Recipients,” American Journal of Transplantation 15, no. 11 (2015): 2970–2977, 10.1111/ajt.13491. 26484839 PMC5292937

[25] K. M. Obeid , S. Sapkota , Q. Cao , et al., “Early *Clostridioides difficile* Infection Characterizations, Risks, and Outcomes in Allogeneic Hematopoietic Stem Cell and Solid Organ Transplant Recipients,” Transplant Infectious Disease: An Official Journal of the Transplantation Society 24, no. 1 (2022): e13720, 10.1111/tid.13720. 34455662

[26] C. Pant , A. Deshpande , M. Desai , et al., “Outcomes of *Clostridium difficile* Infection in Pediatric Solid Organ Transplant Recipients,” Transplant Infectious Disease: An Official Journal of the Transplantation Society 18, no. 1 (2016): 31–36, 10.1111/tid.12477. 26538348

[27] U.S. Department of Health and Human Services , OPTN Policy. 27 Mar. 2025, optn.transplant.hrsa.gov/media/eavh5bf3/optn_policies.pdf.

[28] P. D. Chen , M. K. Tsai , C. Y. Lee , et al., “Gender Differences in Renal Transplant Graft Survival,” Journal of the Formosan Medical Association 112, no. 12 (2013): 783–788, 10.1016/j.jfma.2013.10.011. 24246256

[29] D. Sawinski , J. C. Lai , S. Pinney , et al., “Addressing Sex‐Based Disparities in Solid Organ Transplantation in the United States—A Conference Report,” American Journal of Transplantation 23, no. 3 (2023): 316–325, 10.1016/j.ajt.2022.11.008. 36906294

[30] S. Khumra , J. Chan , K. Urbancic , et al., “Antibiotic Allergy Labels in a Liver Transplant Recipient Study,” Antimicrobial Agents and Chemotherapy 61, no. 5 (2017): e00078–e00117, 10.1128/AAC.00078-17, Published 2017 Apr 24. 28264847 PMC5404540

