# Supplementary material for: The Clinical Impact of Antibiotic Allergy Labels on One‐Year Outcomes of Solid Organ Transplant Recipients
Source: Clin Transplant. 2026 Mar 2;40(3):e70498. doi: 10.1111/ctr.70498 (PMC12954437; doi:10.1111/ctr.70498)
Supplement: Supplementary file 1 — Table S1: Demographics and baseline characteristics with any AAL association. Table S2: Univariate and multivariate analysis of any antibiotic allergy and one‐year primary and secondary outcomes. Table S3: Demographics and baseline characteristics with a BLAL association. Table S4: Univariate and multivariate analysis of beta‐lactam allergy label and one‐year primary and secondary outcomes. Table S5: Trends in cohort factors over time. Appendix A: University of Minnesota Diarrhea/C. Difficile clinical decision support. Appendix B: Details of “other antimicrobial” allergies. [file CTR-40-e70498-s001.docx]

**Supplemental Material for ‘The Clinical Impact of Antibiotic Allergy Labels on 1-year Outcomes of Solid Organ Transplant Recipients’**

**Table of Contents**

- **Supplemental Tables S1-S5**
- **Appendix A- C. Difficle Testing Protocol**
- **Appendix B- Footnote for Table 1, ‘Other Antimicrobial Allergies**

Table S1 Demographics and Baseline characteristics with Any AAL association.

| Patient Characteristic | Overall (N=2373) | No-AAL (N=1801) | Any AAL (N=572) | p-Value |
| --- | --- | --- | --- | --- |
| Age, Mean (SD) | 54.2 years (13.3) | 54.4 (13.2) | 53.7 (13.53) | 0.270 |
| Gender, No. (%) | 906- Female 38.2%  1467- Male 61.8% | 34.3% Female | 50.3% Female | <0.001 |
|  | | | | |
| Kidney | 1080 (45.5%) | 856 (47.5%) | 224 (39.1%) | <0.001 |
| Liver | 599 (25.2%) | 465 (25.8%) | 134 (23.4%) |  |
| Lung | 408 (17.2%) | 260 (14.4%) | 148 (25.8%) |  |
| Heart | 216 (9.1%) | 166 (9.2%) | 50 (8.7%) |  |
| Pancreas | 70 (2.9%) | 54 (3.0%) | 16 (2.8%) |  |
|  | | | | |
| Pre-Transplant Diabetes | 753 (31.7%) | 552 (30.6%) | 201 (35.1%) | 0.050 |
| Pre-Transplant Obesity | 82 (3.5%) | 56 (3.1%) | 26 (4.5%) | 0.114 |
|  | | | | |
| C. Diff < 1 year after transplant | 213 (9.0%) | 143 (7.9%) | 70 (12.2%) | 0.002 |
| Died < 1 year after transplant | 152 (6.4%) | 108 (5.9%) | 44 (7.7%) | 1.69 |
| Graft Loss < 1 year after transplant | 173 (7.3%) | 125 (6.9%) | 48 (8.3%) | 0.268 |
| Index Hospitalization Duration, Mean (SD) | 17.6 days (25.4) | 16.4 days (23.0) | 21.8 days (31.4) | <0.001 |
| Total Inpatient Days After Transplant, Mean (SD) | 25.0 days (34.9) | 23.5 days (32.7) | 29.6 days (40.5) | <0.001 |
| Occurrence of Rejection No ( %) | 602 (25.3%) | 441 (24.4%) | 161 (28.1%) | 0.087 |
|  | | | | |

**Table S1 Legend:**

S.D. – Standard Deviation

D.O.T. – Days of Therapy

Table S2: Univariate and Multivariate analysis of any antibiotic allergy and 1- year primary and secondary outcomes

|  | | | Univariate Analysis | | | Multivariate Analysis | | |
| --- | --- | --- | --- | --- | --- | --- | --- | --- |
| Clinical Outcomes | No-AAL  (N=1801) | Any AAL (N=572) | OR | 95% CI | p-Value | OR | 95% CI | p-Value |
| C diff positive test | 143 (7.9%) | 70 (12.2%) | 1.62 | 1.19-2.19 | 0.002 | 1.450 | 1.060-1.985 | 0.020 |
| Death | 108 (5.9%) | 44 (7.7%) | 1.31 | 0.91-1.89 | 1.169 | 1.169 | 0.797-1.714 | 0.425 |
| Graft Failure | 125 (6.9%) | 48 (8.3%) | 1.22 | 0.86-1.73 | 0.268 | 1.117 | 0.778-1.606 | 0.548 |
| Antibiotic Usage | Average days(S.D.) | Average days(S.D.) | Coefficient | 95% CI | p-Value | Coefficient | 95% CI | p-Value |
| Quinolone- D.O.T | 2.39 (6.11) | 3.67 (6.73) | 1.282 | 0.692-1.872 | <0.001 | 1.210 | 0.616-1.805 | <0.001 |
| Carbapenem- D.O.T | 2.14 (8.33) | 4.67 (10.37) | 2.536 | 1.702-3.370 | <0.001 | 2.480 | 1.644-3.317 | <0.001 |
| Vancomycin- D.O.T | 3.96 (8.30) | 6.02 (11.30) | 2.055 | 1.197-2.913 | <0.001 | 2.105 | 1.243-2.968 | <0.001 |
| Aztreonam-DOT | 0.19 (1.191) | 0.27 (3.87) | 0.074 | -0.164-0.312 | 0.544 | 0.090 | -0.151-0.331 | 0.463 |
| Hospitalization |  | | | | | | | |
| Total days – index hospitalization | 16.39 (23.0) | 21.81 (31.39) | 5.431 | 3.049-7.813 | <0.001 | 5.574 | 3.351-8.156 | <0.001 |
| Total inpatient days in first year | 23.54 (32.77) | 40.56 (34.9) | 6.082 | 2.806-9.359 | <0.001 | 5.835 | 2.576-9.093 | <0.001 |

**Table S2 Legend:**

S.D. – Standard Deviation

D.O.T. – Days of Therapy

Table S3 Demographics and Baseline characteristics with a BLAL association.

| Patient Characteristic | Overall (N=2373) | No BLAL (N=1993) | BLAL (N=380) | p-Value |
| --- | --- | --- | --- | --- |
| Age in years, Mean (SD) | 54.2 years (13.3) | 54.3 (13.2) | 53.7 (13.5) | 0.868 |
| Gender, No. (%) | 906- Female 38.2%  1467- Male 61.8% | 35.9% Female | 49.7% Female | <0.001 |
|  | | | | |
| Kidney | 1080 (45.5%) | 921 (46.2%) | 159 (41.8%) | 0.014 |
| Liver | 599 (25.2%) | 508 (25.5%) | 91 (23.9%) |  |
| Lung | 408 (17.2%) | 319 (16.0%) | 89 (23.4%) |  |
| Heart | 216 (9.1%) | 185 (9.2%) | 31 (8.2%) |  |
| Pancreas | 70 (2.9%) | 60 (3.01%) | 10 (2.6%) |  |
|  | | | | |
| Pre-Transplant Diabetes | 753 (31.7%) | 619 (31.0%) | 134 (35.2%) | 0.118 |
| Pre-Transplant Obesity | 82 (3.5%) | 59 (2.9%) | 23 (6.0%) | 0.005 |
|  | | | | |
| C. Diff < 1 year after transplant | 213 (9.0%) | 168 (8.4%) | 45 (11.8%) | 0.039 |
| Died < 1 year after transplant | 152 (6.4%) | 121 (6.0%) | 31 (8.1%) | 0.137 |
| Graft Loss < 1 year after transplant | 173 (7.3%) | 138 (6.9%) | 35 (9.2%) | 0.131 |
| Index Hospitalization Duration, Mean (SD) | 17.6 days (25.4) | 17.0 days (23.7) | 21.16 (32.7) | 0.004 |
| Total Inpatient Days After Transplant, Mean (SD) | 25.0 days (34.9) | 24.2 days (33.6) | 28.81 (40.9) | 0.020 |
| Occurrence of Rejection No ( %) | 602 (25.3%) | 504 (25.2%) | 98 (25.7%) | 0.847 |

**Table S3 Legend:**

S.D. – Standard Deviation

D.O.T. – Days of Therapy

Table S4: Univariate and Multivariate analysis of Beta-Lactam allergy label and 1- year primary and secondary outcomes

|  | | | Univariate Analysis | | | Multivariate Analysis | | |
| --- | --- | --- | --- | --- | --- | --- | --- | --- |
| Clinical Outcomes | BLAL  (N=1993) | BLAL (N=380) | OR | 95% CI | p-Value | OR | 95% CI | p-Value |
| C diff positive test | 168 (8.4%) | 45 (11.8%) | 1.46 | 1.03-2.06 | 0.039 | 1.343 | 0.939-1.922 | 0.106 |
| Death | 121 (6.0%) | 31 (8.1%) | 1.374 | 0.91-2.07 | 0.137 | 1.345 | 0.877-2.063 | 0.175 |
| Graft Failure | 138 (6.9%) | 35 (9.2%) | 1.364 | 0.925-2.011 | 0.131 | 1.336 | 0.894-1.996 | 0.158 |
| Antibiotic Usage | Average days (S.D.) | Average days (S.D.) | Coefficient | 95% CI | p-Value | Coefficient | 95% CI | p-Value |
| Quinolone- D.O.T | 2.44 (6.11) | 4.01 (7.03) | 1.565 | 0.876-2.254 | <0.001 | 1.482 | 0.790-2.174 | <0.001 |
| Carbapenem- D.O.T | 2.32 (8.57) | 5.01 (10.33) | 2.269 | 1.719-3.671 | <0.001 | 2.659 | 1.684-3.634 | <0.001 |
| Vancomycin- D.O.T | 4.19 (8.76) | 10.92 (9.15) | 1.655 | 0.650-2.660 | 0.001 | 1.692 | 0.686-2.698 | 0.001 |
| Aztreonam-DOT | 0.18 (1.84) | 0.21 (2.53) | 0.196 | -0.082-0.475 | 0.167 | 0.214 | -0.067-0.495 | 0.135 |
| Hospitalization |  | | | | | | | |
| Total days – index hospitalization | 17.03 (23.70) | 21.18 (32.81) | 4.14 | 1.356-6.937 | 0.004 | 4.441 | 1.638-7.245 | 0.002 |
| Total inpatient days in first year | 24.27 (33.57) | 41.05 (34.09) | 4.593 | 0.757-8.429 | 0.019 | 4.248 | 0.449-8.046 | 0.028 |

**Table S4 Legend:**

S.D. – Standard Deviation

D.O.T. – Days of Therapy

Table S5: Trends in Cohort Factors over time

|  | | | | | | | | | | |  |
| --- | --- | --- | --- | --- | --- | --- | --- | --- | --- | --- | --- |
| Year | Total N | Age | MELD  (N=) | C. Difficle PCR positivity | Quinolone DOT | Carbapenem DOT | Vancomycin DOT | Index IPD | Total IPD | Penicillin Allergy Rate | |
| 2012 | 229 | 53.7 | 30.93 (61) | .07 | 2.9389 | 3.9432 | 3.7249 | 13.86 | 24.3712 | .14 | |
| 2013 | 214 | 52.4 | 32.49 (45) | .10 | 2.2664 | 3.1916 | 3.7290 | 15.18 | 20.6729 | .14 | |
| 2014 | 223 | 52.1 | 31.54 (48) | .08 | 3.1480 | 3.3632 | 4.7130 | 18.64 | 25.4619 | .13 | |
| 2015 | 240 | 53.3 | 30.51 (61) | .09 | 3.0417 | 3.3625 | 4.7667 | 17.86 | 26.3792 | .13 | |
| 2016 | 265 | 55.1 | 30.94 (54) | .11 | 2.5774 | 1.9887 | 4.9283 | 17.03 | 26.5057 | .14 | |
| 2017 | 262 | 55.0 | 31.37 (60) | .10 | 2.6298 | 2.3282 | 5.1412 | 17.01 | 25.2137 | .14 | |
| 2018 | 209 | 56.3 | 30.43 (56) | .10 | 3.1770 | 2.3301 | 5.5167 | 22.57 | 27.4976 | .14 | |
| 2019 | 278 | 54.5 | 29.55 (73) | .08 | 2.5072 | 2.4424 | 4.3058 | 18.02 | 24.8849 | .13 | |
| 2020 | 213 | 55.1 | 28.30 (56) | .10 | 2.5540 | 2.0423 | 4.7653 | 18.49 | 26.2113 | .15 | |
| 2021 | 240 | 54.5 | 28.74 (85) | .07 | 2.2000 | 2.6875 | 2.9750 | 18.67 | 22.7500 | .13 | |
| Total | 2373 | 54.2 | 30.33 (599) | .09 | 2.6949 | 2.7501 | 4.4576 | 17.69 | 25.0114 | .14 | |

**Table S5 Legend:**

N – Number

DOT – Days of therapy

IPD – Inpatient days

Appendix A: University of Minnesota Diarrhea / C. Difficile Clinical Decision Support

In addition to various unit and departmental diagnostic stewardship education efforts ,when a provider attempts to order Clostridioides Difficle testing in our EMR they are met with a decision support tool as follows:

Step 1: Confirm clinical diagnosis of diarrhea- must have at least one of the following

1. Does the patient have 3 or more liquid stools (and/or 500mL of liquid stool output) in a 24-hour period?
2. Has the patient been off laxatives/stool softeners for at least 48 hours?

Once this has been completed the provider is taken to the next step

Step 2: Risk Stratify the diarrheal patient

Review of Sodium, Potassium, Creatinine, Anion Gap, Carbon Dioxide and WBC count from recent lab draws and determine if Low Risk or High Risk based on provider judgement.

IF Low Risk the following reminder is given:

1. Many hospitalized patients have diarrhea, but CDI is the cause for diarrhea only about 15% of the time. In patients without high-risk features, it is important to consider alternative causes of diarrhea.
2. Focus on identifying, and removing, possible offending agents
3. [Medications associated with diarrhea - UptoDate](https://www.uptodate.com/contents/image?imageKey=GAST/71449&topicKey=PEDS%2F5877&search=Medications+associated+with+diarrhea&source=outline_link&selectedTitle=1~150) Link
4. If concerns for diarrhea due to inflammatory bowel disease, exogenous pancreatic insufficiency, osmotic diarrhea, cystic fibrosis, or bariatric surgery, consider nutrition services consult (Note: all patients on tube feeds are followed by Nutrition Services)
5. Consider Symptomatic treatment
6. Test for infectious causes of diarrhea if non-infectious causes seem unlikely

Then the provider is offered options

1. For symptomatic treatment such as loperamide
2. Test for CDI if indicated with a reminder that “ Standard criteria for C. difficile testing: three or more loose/watery stools in a 24hr period that cannot be attributable to another cause plus at least one symptoms of CDI (abdominal pain, fever, or elevated WBC) “

If High Risk the provider is prompted to:

1. Test for CDI if indicated with a reminder that “ Standard criteria for C. difficile testing: three or more loose/watery stools in a 24hr period that cannot be attributable to another cause plus at least one symptoms of CDI (abdominal pain, fever, or elevated WBC) “
2. Adress other issues such, check whole blood lactic acid, abdominal imaging, electrolyte replacement.

Appendix B – Details of “Other Antimicrobial” Allergies

Aminoglycosides Including; Amikacin, Colistin, Fosfomycin, Gentamicin, Neomycin, Polymyxin B, Tobramycin.

Atovaquone

Aztreonam

Bacitracin

Clindamycin

Clavulanic-Acid

Dapsone

Daptomycin

Ethambutol

Imipenem and Imipenem/Cilastatin

Isoniazid

Linezolid

Metronidazole

Meropenem

Nitrofurantoin

Rifampin

Sulbactam

Tigecycline
